# Long‐term outcomes of metastasis‐directed stereotactic body radiation therapy in metastatic nasopharyngeal carcinoma

**DOI:** 10.1002/cam4.6764

**Published:** 2023-12-26

**Authors:** Jiangping Yang, Wenjun Liao, Shitong Su, Ni Zeng, Shichuan Zhang, Jinlan He, Nianyong Chen

**Affiliations:** ^1^ Department of Head and Neck Oncology and Department of Radiation Oncology, Cancer Center and State Key Laboratory of Biotherapy, West China Hospital Sichuan University Chengdu China; ^2^ Department of Radiation Oncology, Radiation Oncology Key Laboratory of Sichuan Province Sichuan Clinical Research Center for Cancer, Sichuan Cancer Hospital & Institute, Sichuan Cancer Center, Affiliated Cancer Hospital of University of Electronic Science and Technology of China Chengdu China

**Keywords:** distant metastasis, nasopharyngeal carcinoma, PD‐1 inhibitors, stereotactic body radiation therapy, survival

## Abstract

**Background:**

The study aims to evaluate the outcomes of metastasis‐directed stereotactic body radiation therapy (SBRT) in metastatic nasopharyngeal carcinoma (mNPC).

**Methods:**

We reviewed all SBRT conducted in patients with mNPC in our institution between 2013 and 2022. Systemic therapy was performed with chemotherapy with or without anti‐programmed death‐1 (PD‐1) therapy. Local treatment delivered with ablative purpose in stereotactic setting with dose/fraction ≥5 Gy was evaluated. Kaplan–Meier analyses were used to determine the rates of local control (LC), progression‐free survival (PFS), and overall survival (OS). Univariate and multivariate analyses were performed by Cox regression.

**Results:**

A total of 54 patients with 76 metastatic sites receiving SBRT were analyzed. Median follow‐up was 49 months. The 3‐year LC, PFS, and OS rates were 89.1%, 29.4%, and 57.9%, respectively. Adding a PD‐1 inhibitor to SBRT tended to prolong median OS (50.1 vs. 32.2 months, *p* = 0.068). Patients receiving a biological effective dose (BED, α/β = 10) ≥ 80 Gy had a significantly longer median OS compared to those who received a lower dose (not reached vs. 29.5 months, *p* = 0.004). Patients with oligometastases (1–5 metastases) had a better median OS (not reached vs. 29.5 months, *p* < 0.001) and PFS (34.3 vs. 4.6 months, *p* < 0.001). Pretreatment EBV‐DNA and maintenance therapy were also significant predictors for OS.

**Conclusions:**

Metastatic NPC patients could benefit from metastases‐directed SBRT in combination with systemic therapy.

## 1 INTRODUCTION

Nasopharyngeal carcinoma (NPC) is rare in Western countries but is endemic in Southeast Asia and Southern China.^1^ With the development of intensity‐modulated radiotherapy (IMRT) and comprehensive treatment strategy including chemotherapy and immunotherapy, the local control (LC) of NPC has been greatly improved, and distant metastasis has become the leading cause of treatment failure and patient mortality.^2^, ^3^ Approximately 6%–15% of patients show distant metastasis at first diagnosis, and 20%–30% of patients with locally advanced NPC develop metastatic disease after initial radical chemoradiotherapy.^2^, ^4^, ^5^ The survival of patients with metastatic NPC (mNPC) is poor, with a median overall survival (OS) of 12–30 months despite multidisciplinary therapies.^1^, ^6^, ^7^

Systemic chemotherapy with or without immunotherapy remains the main treatment for mNPC. Local treatment is also recommended for selected patients with limited metastases or a low tumor burden.^8^, ^9^ Moreover, several studies have demonstrated that patients with mNPC can benefit from local radiotherapy, either alone or in combination with chemotherapy.^7^, ^8^, ^10^, ^11^, ^12^ However, the optimal radiation modality for metastases remains to be elucidated.

Stereotactic body radiation therapy (SBRT) is a promising local treatment modality. In contrast to conventional fractionated radiotherapy, SBRT precisely delivers highly conformal large doses of radiation in several fractions to target volumes, enabling tumor ablation while minimizing dose to adjacent critical organs at risk (OARs).^13^ Stereotactic body radiation therapy has been associated with optimal control of oligometastatic disease even with curative intent and lower toxicity,^14^, ^15^ particularly in lung, liver, bone, or brain metastases,^16^, ^17^ with LC rates ranging from 70% to 90%.^18^

Although local radiotherapy is now recommended as an option for local treatment of mNPC, there is limited evidence on the outcomes of SBRT in patients who are simultaneously receiving systemic therapy. The aim of this study is to investigate the survival benefit of SBRT delivered to distinct metastases combined with systemic therapy for mNPC patients, providing more information on the value of SBRT for metastases in mNPC.

## 2 METHODS AND MATERIALS

### 2.1 Study population

**FIGURE 1:**
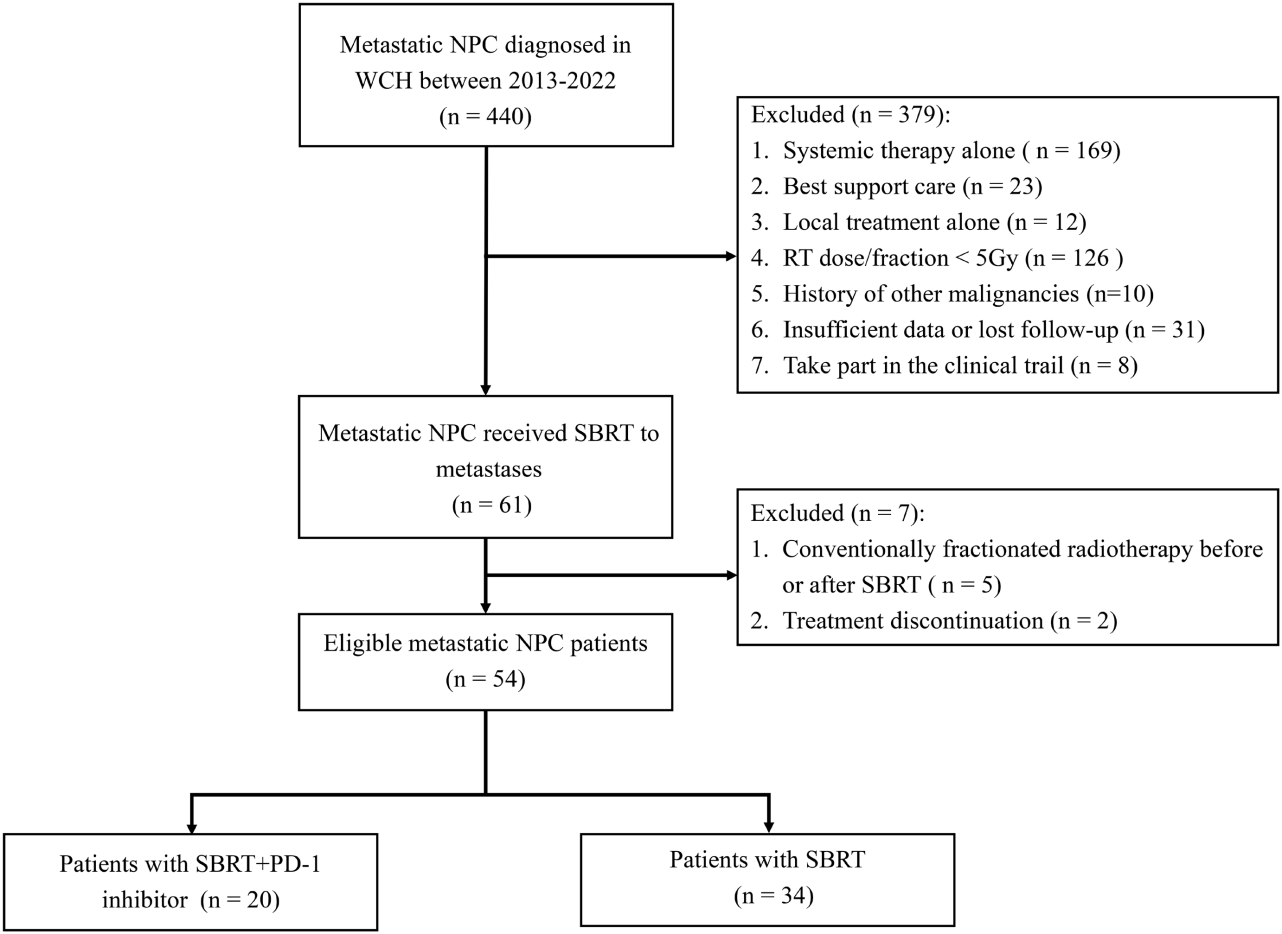
Flow diagram of study selection process.

Data of patients with mNPC treated at the West China Hospital of Sichuan University from January 2013 and February 2022 were collected. The eligibility criteria were as follows: (1) histologically diagnosed NPC; (2) distant metastasis confirmed by radiographic and/or histological evidence; (3) SBRT for metastases performed with doses per fraction ≥5 Gy; (4) at least one cycle of systemic chemotherapy; (5) with or without programmed death‐1 (PD‐1) inhibitors; (6) Eastern Cooperative Oncology Group (ECOG) performance score ≤1; (7) no prior local treatment to the metastatic sites. The exclusion criteria were as follows: (1) metastases treated with surgery, radiofrequency, or conventionally fractionated radiotherapy; (2) a history of prior or concurrent malignancies; and (3) insufficient clinical data or loss of follow‐up. All patients were restaged based on the TNM staging system by the American Joint Committee on Cancer (AJCC) 8th edition. The selection process is presented in Figure 1.

### 2.2 Diagnosis and treatments

The definition of metastasis should be confirmed by biopsy or at least one of the following imaging: contrast‐enhanced chest computed tomography (CT), abdominal ultrasound, contrast‐enhanced abdominal magnetic resonance imaging (MRI), CT, or positron emission tomography/computed tomography (PET/CT). Bone metastases were screened using emission computed tomography of whole‐body bones, and the suspected lesions were further confirmed with contrast‐enhanced CT or MRI, PET/CT, or pathological diagnosis.

The treatment regimens were extracted from the electronic medical records. Most patients received platinum‐based chemotherapy, including GP (gemcitabine plus cisplatin), TP/TPF (docetaxel or paclitaxel plus cisplatin with or without 5‐flurouracil), PX (cisplatin plus capecitabine), or capecitabine alone. All above regimens were administered every 3 weeks. Some patients also received PD‐1 inhibitors. Detailed information on chemotherapy regimens and PD‐1 inhibitors is listed in Table S1.

Due to the lack of established recommendations on SBRT in mNPC, the target lesions and timing of SBRT were determined individually by a multidisciplinary team based on the characteristics of each case including ECOG score, symptoms, previous treatments, response to treatment, and predicted survival.^19^ Stereotactic body radiation therapy was applied every other day to irradiate all the visible lesions in patients with oligometastases (1–5 metastases) and focused on the main metastatic lesions in the multiple metastatic setting or lesions that failed to respond to chemotherapy. For patients with lung or liver metastases, four‐dimensional computed tomography (4DCT) scan was used to measure respiratory motion, and the diaphragm's motion was limited to 1 cm by using abdominal compression or an active breathing control was applied for breath hold. Volumetric modulated arc therapy or IMRT was used.

Gross tumor volume (GTV) was defined as the visible gross tumor mass in the contrast‐enhanced planning CT. The planning target volume (PTV) was created by adding an additional 0.5–1.0 cm to each GTV. And the coverage of the PTV was intended to exceed 95% of the prescribed dose. Published dose constraints for OAR in SBRT were used.^20^ To correlate irradiated doses with clinical outcomes, the biological effective dose (BED) was calculated by assuming an α/β ratio of 10 Gy for the metastases and using the linear‐quadratic model:

$$\begin{array}{c} {\mathrm{BED}}_{10}\left(\mathrm{Gy}\right)=\text{fractional dose}\\ \;\times \text{number of fractions}\;\left(1+\frac{\text{fractional dose}}{\frac{\alpha }{\beta }}\right). \end{array}$$

### 2.3 Follow‐up and endpoints

Patients were routinely assessed by chest CT, abdominal CT/MRI, and bone scans every two or three cycles of systemic treatment to evaluate the effect. Patients were assessed by whole blood count, liver and kidney function, chest CT, abdominal CT/MRI, bone scan, and test for any additional newly suspected sites of progression every three cycles of treatment. After completion of all treatment, patients were routinely monitored every 3 months during the first 3 years, every 6 months from years 4–5, and then annually until death or loss to follow‐up. The primary outcome was LC, which was described as free from local progression at SBRT‐treated sites. OS was calculated from the initiation of first‐line palliative therapy to the last follow‐up or death from any cause, whichever occurred first. Progression‐free survival (PFS) was calculated from SBRT to disease progression or death. Patients who were alive at the last follow‐up were censored on that day. Clinical response to SBRT was classified as complete response (CR), partial response (PR), stable disease (SD), or progressive disease (PD). Treatment evaluation was based on Response Evaluation Criteria in Solid Tumors 1.1 (RECIST 1.1).

### 2.4 Statistical analysis

Statistical analyses were performed with SPSS software version 25.0 and R version 4.2.3. The Kaplan–Meier method was used to analyses the median follow‐up time, LC, PFS, and OS. Univariate and multivariate analyses were carried out using Cox regression. Statistical significance was defined as a two‐side *p*‐value < 0.05.

## 3 RESULTS

### 3.1 Patient characteristics and treatment

A total of 54 patients were included in the analyses. Table 1 shows the baseline characteristics. The median age of patients was 46 years (range: 22–64 years), and the male‐to‐female ratio was 4.4:1. Patients had ECOG score <1 in 72.2% of cases. Seventeen patients (31.5%) had synchronous metastasis, 94.1% of which had T3‐4 and N2‐3 disease. The most frequent metastatic sites were the lung (*n* = 34, 63.0%), liver (*n* = 25, 46.3%), and bone (*n* = 15, 27.8%; Table 1). In addition, 30 patients (55.6%) presented with single metastatic site. Twenty‐nine patients had no more than five metastases, 23 (79.3%) of which received SBRT to all metastases.

**TABLE 1:** Patient characteristics.

Characteristic	Overall (*n* = 54)
	No. of patients (%)
Age (y), median (range)	46 (22–64)
Sex	
Male	44 (81.5)
Female	10 (18.5)
ECOG performance status	
0	39 (72.2)
1	15 (27.8)
Plasma EBV‐DNA (copies/mL)^a^	
≤10^3^	36 (66.7)
>10^3^	16 (29.6)
Missing^b^	2 (3.7)
Metastatic organs	
Lung	34 (63.0)
Liver	25 (46.3)
Bone	15 (27.8)
Distant lymph node	9 (16.7)
Metastatic sites	
Single	30 (55.6)
Multiple	24 (44.4)
Total metastases	
≤5	29 (53.7)
>5	25 (46.3)
Synchronous metastasis	17 (31.5)
Median time from metastasis diagnosis to SBRT (months, range)	6.1 (0.6–24.9)
No. of lines of SBRT treatment	
1	41 (75.9)
2 or more	17 (31.5)
PD‐1 inhibitors	20 (37.0)
No. of lines of PD‐1 treatment	
1	15 (27.8)
2 or more	8 (14.8)
Maintenance therapy	21 (38.9)
Cycles of systemic therapy (range)	5 (1–10)

*Note*: Data are presented as *n* (%) unless otherwise indicated.

Abbreviations: EBV, Epstein–Barr Virus; ECOG, Eastern Cooperative Oncology Group; No., number; PD‐1, programmed death‐1; SBRT, stereotactic body radiation therapy.

^a^ Plasma EBV‐DNA detected before treatment.

^b^ EBV‐DNA detection was refused by these patients.

The timing of chemotherapy and SBRT is shown in Table S2. Overall, the median time from the diagnosis of metastasis to chemotherapy and SBRT was 0.5 months (IQR, 0.2–1.1) and 6.1 months (IQR: 2.6–11.0 months), respectively. The median time from the initiation of first‐line chemotherapy to SBRT was 5.5 months (IQR: 1.8–9.7 months). Forty‐one (75.9%) patients received SBRT in first‐line therapy, 14 in the second‐line, 2 in the third‐line, and 1 in the fourth‐line.

All patients underwent systemic chemotherapy with one cycle or more (median: 5). Regarding the chemotherapy regimens, 26 (48.1%), 12 (22.2%), and 9 (16.7%) patients received GP, TPF/TP, and PX, respectively. Of all patients, 20 patients (37.0%) received a PD‐1 inhibitor, of which 15 received in the first line, 5 in second line, 1 in third line, and 2 in the fourth line palliative chemotherapy. Maintenance therapy was administered in 38.9% (*n* = 21) of patients, which included a PD‐1 inhibitor alone (*n* = 8), capecitabine alone (*n* = 4), an antiangiogenic drug alone (*n* = 2), and a combination of capecitabine with either a PD‐1 inhibitor (*n* = 6) or an antiangiogenic drug (*n* = 1; Table S1).

### 3.2 Response to SBRT

**FIGURE 2:**
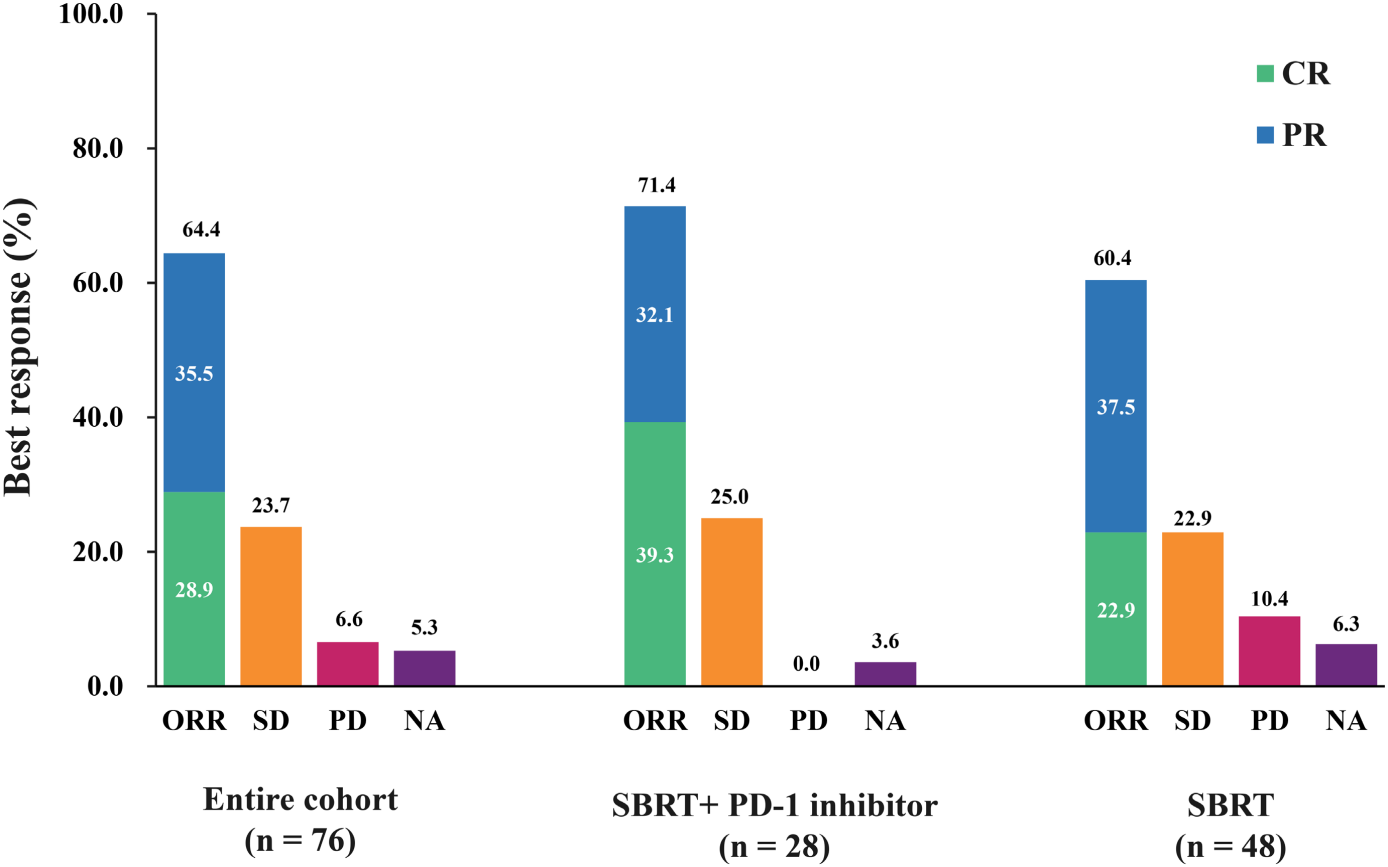
Summary of best response to treatment.

Stereotactic body radiation therapy was applied to a total of 76 sites, 39 (51.3%) of which were in the lungs and 27 (35.5%) in the liver (Table 2). The median number of irradiated sites per patient was one (range: 1–5). Fractionation schemes varied, and the most frequently prescribed dose was 48 Gy in 4–8 fractions, accounting for 44.7% (*n* = 34) of all cases (Table 2). Stereotactic body radiation therapy was performed with a median dose of 48 Gy (range: 9–56 Gy) in a median fractionation of 4 (range: 1–10), resulting in a median BED_10_ of 80 Gy (range: 17 Gy–106 Gy) (Table 3). The rate of CR, PR, SD, and PD after SBRT were 28.9% (*n* = 22), 35.5% (*n* = 27), 23.7% (*n* = 18), and 6.6% (*n* = 5), respectively. The ORR of irradiated sites was 64.4% (Figure 2). Seven sites (9.2%), four located in liver and three in lung, developed in‐filed progression with a median time of 4.1 months (range: 1.6–14.7 months) after SBRT. Detailed prescribed doses according to localization of metastases are reported in Table 3.

**TABLE 2:** Summary of SBRT treatment characteristics.

Characteristic	No. (%)
Treated sites	
Median (range)	1 (1–5)
Single	39 (72.2)
Multiple	15 (27.8)
Treated metastases	
1	42 (55.3)
2	17 (22.4)
3	7 (9.2)
4	3 (3.9)
5	2 (2.6)
≥6	5 (6.6)
Localization of treated metastases	
Lung	39 (51.3)
Liver	27 (35.5)
Bone	7 (9.2)
Distant lymph node	3 (3.9)
Dose‐fractionation schemes (Gy/fractions)	
9–42/1–7	31 (40.8)
48/4–8	34 (44.7)
50–56/7–10	11 (14.5)
Response to SBRT	
Objective response	49 (64.4)
Complete response	22 (28.9)
Partial response	27 (35.5)
Stable disease	18 (23.7)
Progression disease	5 (6.6)
Missing data or could not be evaluated	4 (5.3)

Abbreviations: No., number; SBRT, stereotactic body radiation therapy.

**TABLE 3:** SBRT schedule according to localization of metastatic disease.

Data	Median (range)
SBRT schedules of all sites	
Total dose, Gy	48 (9–56)
Fractions number	4 (1–10)
Dose per fraction, Gy	8 (5–12)
BED^a^, Gy	80 (17–106)
Lung (*n* = 39)	
Total dose, Gy	48 (9–56)
Fractions number	4 (1–9)
Dose per fraction, Gy	12 (6–12)
BED, Gy	101(17–106)
Liver (*n* = 27)	
Total dose, Gy	48 (18–56)
Fractions number	5 (3–10)
Dose per fraction, Gy	8 (5–12)
BED, Gy	79 (29–106)
Bone (*n* = 7)	
Total dose, Gy	42 (16–54)
Fractions number	6 (2–8)
Dose per fraction, Gy	7 (6–8)
BED, Gy	71 (29–86)
Distant lymph nodal (*n* = 3)	
Total dose, Gy	48 (30–56)
Fractions number	5 (4–8)
Dose per fraction, Gy	7 (6–12)
BED, Gy	95 (48–106)

Abbreviations: BED, biological effective dose; SBRT, stereotactic body radiation therapy.

^a^ BED was calculated by assuming an α/β ratio of 10 Gy.

### 3.3 Local control and survival

**FIGURE 3:**
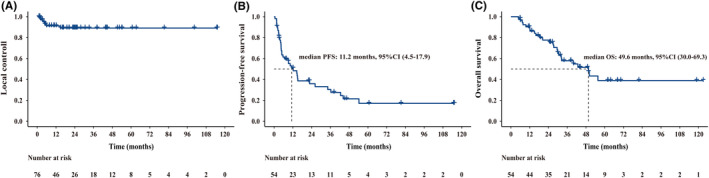
Kaplan–Meier curves for local control (A), progression‐free survival (B), and overall survival (C).

The median follow‐up after first‐line therapy and SBRT was 49.0 months (range: 4.8–123.0 months) and 39.0 months (range: 1.9–115.3 months), respectively. At the last follow‐up, 38 patients (70.4%) experienced distant disease progression at a median of 6.4 months (range: 0.6–54.3 months), and 24 patients (44.4%) died. The LC rates of SBRT‐treated metastases after 1 year and 3 years were 91.2% and 89.1%, respectively (Figure 3A). The 1‐ and 3‐year PFS rates were 49.1% and 29.4%, respectively, and the 1‐ and 3‐year OS rates were 90.3% and 57.9%, respectively (Figure 3B,C).

At present, PD‐1 inhibitor have been recognized as a key drug in recurrent/ metastatic NPC. Therefore, we investigated whether the use of PD‐1 inhibitors affected the survival of patients with mNPC. Patients were divided into two groups. One included 20 patients with 28 sites treated with SBRT and chemotherapy plus a PD‐1 inhibitor, while the other included 34 (63.0%) patients treated with SBRT and chemotherapy. For patients receiving PD‐1 inhibitors, the rate of CR, PR, and SD of irradiated sites was 39.3% (*n* = 11), 32.1% (*n* = 9), and 25.0% (*n* = 7), respectively, leading to an ORR of 71.4% (Figure 2). Two patients suffered from a local relapse 4.4 months and 3.7 months after SBRT, respectively. The addition of PD‐1 inhibitors to SBRT tended to prolong median OS (50.1 vs. 32.2 months, *p* = 0.068), although the difference was not statistically significant (Figure S1).

**FIGURE 4:**
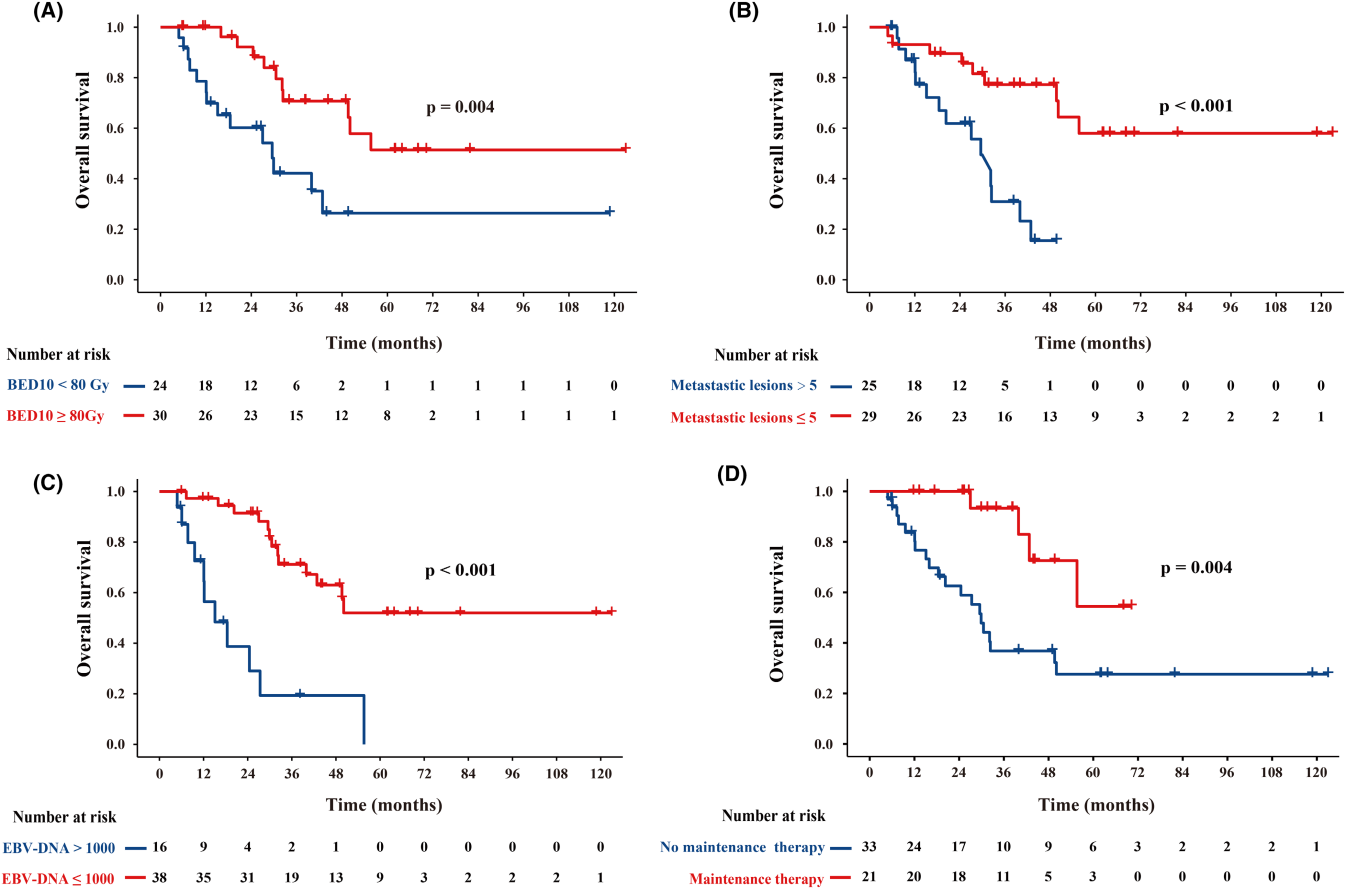
Kaplan–Meier curves for overall survival stratified by BED (A), metastatic lesions (B), pretreatment plasma EBV‐DNA (C), and maintenance therapy (D).

To investigate whether radiation dose influences OS and PFS, the median BED_10_ value of 80 Gy was used to divide patients into two groups. Patients receiving a BED_10_ ≥ 80 Gy achieved a significant longer median OS (not reached vs. 29.5 months, *p* = 0.004; Figure 4A) and PFS (26.2 vs. 4.5 months, *p* = 0.002) compared to those who receiving a BED_10_ < 80 Gy. In addition, the median OS was significantly longer for patients with 1–5 metastases (not reached vs. 29.5 months, *p* < 0.001) or plasma EBV‐DNA ≤1000 copies/mL before treatment (not reached vs. 15.1 months, *p* < 0.001; Figure 4B,C). Moreover, maintenance therapy substantially improved the median OS compared to observation (not reached vs. 29.9 months, *p* = 0.004; Figure 4D).

### 3.4 Univariate and multivariate analysis

In univariate analysis, the ECOG score, pretreatment plasma EBV‐DNA, BED_10_, the number of metastases, and bone metastasis were associated with both OS and PFS (*p* < 0.05; Table 4 and Table S3). On multivariate analysis, BED_10_ ≥ 80 Gy (HR 0.10, 95% CI 0.02–0.52, *p* = 0.006), oligometastases (HR 0.19, 95% CI 0.05–0.66, *p* = 0.009), plasma EBV‐DNA ≤1000 copies/mL (HR 0.06, 95% CI 0.02–0.24, *p* < 0.001), and maintenance therapy (HR 0.07, 95% CI 0.02–0.28, *p* < 0.001) remained significant predictors for longer OS. As for PFS, ECOG score, sex, plasma EBV‐DNA, oligometastases, and time from the diagnosis of metastasis to SBRT were demonstrated to be related to PFS.

**TABLE 4:** Univariate and multivariate analysis of overall survival.

Factor	Univariate analysis			Multivariate analysis		
	HR	95% CI	*p* Value	HR	95% CI	*p* Value
Sex (male vs. female)	1.36	0.46–3.98	0.578			
Age (≥46 vs. <46 years)	1.28	0.57–2.90	0.555			
ECOG (1 vs. 0)	4.58	1.90–11.09	0.001			
EBV‐DNA (≤1000 vs. >1000 copies/mL)	0.17	0.07–0.39	<0.001	0.06	0.02–0.24	<0.001
PD‐1 inhibitors (yes vs. no)	0.41	0.15–1.10	0.077			
Synchronous metastasis (yes vs. no)	1.39	0.60–3.26	0.443			
Metastatic sites (single vs. multiple)	0.48	0.21–1.11	0.088			
Lung metastasis (yes vs. no)	1.24	0.49–3.12	0.655			
Liver metastasis (yes vs. no)	0.99	0.44–2.24	0.983			
Bone metastasis (yes vs. no)	2.65	1.12–6.26	0.027	3.23	1.15–9.06	0.026
Oligometastases (yes vs. no)^a^	0.22	0.09–0.55	0.001	0.19	0.05–0.66	0.009
Dose/fraction (≥8 vs. <8 Gy)	0.61	0.26–1.43	0.253			
Total dose (≥48 vs. <48 Gy)	0.45	0.20–1.03	0.059	0.15	0.03–0.69	0.015
BED (≥80 vs. <80 Gy)	0.31	0.13–0.72	0.006	0.10	0.02–0.52	0.006
Time from metastasis diagnosis to SBRT (≤6 vs. >6 months)	0.60	0.26–1.38	0.230			
Cycles of systemic therapy (≤5 vs. >5 cycles)	0.41	0.18–0.97	0.042			
Maintenance therapy (yes vs. no)	0.23	0.08–0.69	0.008	0.07	0.02–0.28	<0.001

Abbreviations: BED, biological effective dose; EBV, Epstein–Barr Virus; ECOG, Eastern Cooperative Oncology Group; HR, Hazard Ratio; PD‐1, programmed death‐1; SBRT, stereotactic body radiation therapy.

^a^ Oligometastases was defined as a limited metastatic spread (between 1 and 5 metastases) and low tumor burden.

## 4 DISCUSSION

Although there is an increasing interest in metastasis‐directed SBRT for mNPC, evidence is still under investigation. To our knowledge, this is the first study to evaluate the long‐term outcomes of metastasis‐directed SBRT combined with systemic treatment on prognosis of patients with mNPC. The result showed that metastases‐directed SBRT brought a considerable survival benefit with a 3‐year LC, PFS, and OS rate of 89.1%, 29.4%, and 57.9%, respectively. Second, adding a PD‐1 inhibitor to chemotherapy and SBRT showed a tendency of longer OS. Third, oligometastases, BED_10_ ≥ 80 Gy, and maintenance therapy were significant predictors for better OS.

Systemic chemotherapy remains the standard care of mNPC. However, patients had an unsatisfactory outcome when treated with firstline GP regimen, with a 1‐year PFS rate of 20.0% and median OS of 22.1 months. The addition of PD‐1 inhibitors further improved the efficacy, with a median PFS of 9.6–11.7 months, and has been recommended as a new first‐line regimen.^21^, ^22^, ^23^ Furthermore, several studies have demonstrated that local radiotherapy added to chemotherapy significantly improved the survival of patients with mNPC when compared with chemotherapy alone,^7^, ^10^, ^11^, ^24^ with 3‐year OS increased to 48.8–61.2% from 25.9–47.8%. However, conventional fractionated radiotherapy has been adopted in most studies. Theoretically, SBRT could bring more benefits compared with conventional radiotherapy because of its potential biological effects, shorter treatment time, less toxicity, and flexible matching with systemic therapy. In fact, accumulating evidences suggests that SBRT could provide superior LC for metastases, with 2‐year LC rate of 83.6% for lung cancer, 82.2% for breast cancer, 81.0% for prostate cancer, and 59.3% for rectal cancer.^25^, ^26^ In our study, the 3‐year LC, PFS, and OS rates for patients with mNPC who received SBRT in combination of systemic chemotherapy were 89.1%, 29.4%, and 57.9%, with a median OS of 49.6 months. The results supported that SBRT is an effective local treatment for mNPC.

Apart from local benefit, SBRT may trigger systemic effects, reduce tumor‐induced immune suppression,^27^, ^28^, ^29^ and enhance the efficacy of PD‐1 immunotherapy.^30^ Combining SBRT with a PD‐1 inhibitor can improve clinical response rates and prolong survival in both local and metastatic disease.^31^, ^32^ In a recent study, seven cases of recurrent/metastatic NPC were treated with SBRT in combination with a PD‐1 inhibitor. Six out of the seven patients achieved ORR (85.7%), with a 2‐year OS rate of 71.0% and a median OS of 30.8 months.^33^ In this study, 37.0% of patients (*n* = 20) received concurrent administration of a PD‐1 inhibitor with SBRT, and 14 patients (25.9%) continued PD‐1 inhibitors as maintenance therapy. Although not statistically significant, adding a PD‐1 inhibitor to SBRT showed a tendency to extend the clinical benefit of mNPC. In addition, 71.4% of irradiated lesions achieved ORR (CR or PR). A possible explanation for the results was the small sample size, which required a large effect size for the results to be statistically significant. Furthermore, the good response in patients treated with SBRT plus chemotherapy would cover the possible additive or synergistic benefit of PD‐1 inhibitors. Further clinical trials are needed to investigate the survival benefits of SBRT combined with PD‐1 inhibitors.

Metastasis is a sequential, multistep process. Therefore, it's possible that certain distant metastases are curable. Oligometastatic disease is an intermediate state between localized primary tumors and widespread metastases.^34^ It is characterized by a limited number of metastases (1–5 metastases), organ‐specificity, modest biological invasion, and low tumor burden. Stereotactic body radiation therapy is widely employed to treat oligometastases. Lack of evidence on SBRT for oligometastatic NPC was the primary motivation for the current study, where promising survival benefits were observed. Stereotactic body radiation therapy was demonstrated to be associated with more favorable median OS and PFS for patients with oligometastatic NPC. These encouraging survival outcomes suggested that patients with oligometastatic NPC do benefit from appropriate local SBRT plus systemic therapy. In addition, our study suggested that plasma EBV‐DNA ≤ 1000 copies/mL before treatment was a positive prognostic factor of prolonged OS in mNPC patients compared to those with higher EBV‐DNA levels, which was consistent with previous studies.^35^, ^36^, ^37^ EBV‐DNA is a crucial biomarker of tumor burden in NPC and has been recommended for tumor staging and treatment response monitoring.^38^, ^39^, ^40^ Therefore, for patients with favorable prognostic characteristics and a small tumor burden (oligometastases and/or a low level of pretreatment EBV‐DNA), a curative strategy consisting of systemic therapy and metastases‐directed SBRT may be an optimal therapeutic approach.

At present, the optimal dose and fractionation of SBRT in metastases are poorly characterized. Several studies indicated that ablative doses of BED₁₀ ≥ 100 Gy are correlated with long‐term LC.^41^, ^42^, ^43^ In clinical practice, BED₁₀ doses above 100 Gy are regularly used to treated lung and liver metastases, while lower SBRT doses are used to treated metastases in other organs.^44^ Stereotactic body radiation therapy of metastases at doses below the ablative threshold of BED₁₀ 100 Gy is supported by prospective studies, particularly in the management of oligometastases when SBRT is combined with efficacious systemic therapy. In terms of mNPC, some studies suggested that patients with mNPC receiving chemotherapy plus local radiotherapy with a BED_10_ ≥ 60 Gy had a better outcome than those receiving a BED_10_ < 60 Gy.^7^, ^10^ However, most of those patients received conventional fractionated radiotherapy. In our analysis, we found that patients treated with chemotherapy who receiving a BED_10_ ≥ 80 Gy had a better OS than those who were prescribed a BED_10_ < 80 Gy. In this context, one of the critical questions is whether ablative radiation doses are required or if conventional fractionated radiotherapy could lead to superior efficacy when combined with effective systemic therapy. Further studies are required to figure it out.

Maintenance therapy is a treatment approach that keeps administering drugs after the tumor burden has been reduced to the minimum, with the goal of delaying progression and extending OS. This strategy has been proven successful in the treatment of metastatic lung,^45^ breast,^46^ and colorectal cancers.^47^ Maintenance therapy with capecitabine has also shown promising outcomes in the treatment of mNPC.^48^ In our study, 21 patients received maintenance therapy with PD‐1 inhibitors, capecitabine, or antiangiogenic agent alone or in combination after SBRT. The results suggested that patients receiving maintenance therapy had a significantly longer median OS compared with observation. Besides capecitabine, studies have demonstrated that PD‐1 inhibitors and antiangiogenic agents may also be promising maintenance options for recurrent or metastatic NPC,^49^, ^50^, ^51^ which were used as maintenance therapy in clinical practice. However, due to limited evidences, it is worth studying the efficacy and safety of chemotherapy, PD‐1 inhibitors, or antiangiogenic maintenance therapy alone or in combination with SBRT.

This study has several limitations as well. First, the study was limited in its statistical power due to its single‐center design and relatively small sample size. Second, selection bias should be considered because of the retrospective nature of the study. Patients who treated with SBRT may be the most suitable candidates, and the dose and fractionation of SBRT were determined by a multidisciplinary team. Third, the timing of systemic therapies and SBRT delivery differed depending on the physicians' choices, which potentially influenced the treatment response and survival outcomes.

In conclusion, SBRT is an effective treatment option for mNPC patients. For patients with low tumor burden or oligometastases, a higher dose of SBRT to metastases combined with systemic therapy is recommended. The effect of adding a PD‐1 inhibitor to SBRT plus chemotherapy remains unknown. Further well‐designed studies are warranted to explore the clinical benefit and predictive biomarkers of SJBRT combined with PD‐1 inhibitors.

## AUTHOR CONTRIBUTIONS

**Jiangping Yang:** Data curation (equal); writing – original draft (equal); writing – review and editing (equal). **Wenjun Liao:** Resources (equal); writing – review and editing (equal). **Shitong Su:** Data curation (equal); software (equal). **Ni Zeng:** Data curation (equal); investigation (equal). **Shichuan Zhang:** Data curation (equal); software (equal). **Jinlan He:** Conceptualization (equal); project administration (equal); writing – review and editing (equal). **Nianyong Chen:** Conceptualization (equal); supervision (equal); writing – review and editing (equal).

## FUNDING INFORMATION

This research did not receive any specific grant from funding agencies in the public, commercial, or not‐for‐profit sectors.

## CONFLICT OF INTEREST STATEMENT

The authors declare that they have no potential financial competing interests.

## ETHICS STATEMENT

The study was approved by the Ethics Committee of West China Hospital, Sichuan University (approval no. 2021‐1085) and was conducted in accordance with the 1964 Helsinki Declaration and its later amendments. Informed consent was waived by our Institutional Review Board because of the retrospective nature of our study.

## CONSENT

Informed consent was waived by the Institutional Review Board because of the retrospective nature of our study.

## Supporting information


Appendix S1.

## Data Availability

All data generated or analyzed during this study are included in this published article. Further data are available upon reasonable request.
