# Supplementary material for: Long‐term outcomes of metastasis‐directed stereotactic body radiation therapy in metastatic nasopharyngeal carcinoma
Source: Cancer Med. 2023 Dec 26;13(1):e6764. doi: 10.1002/cam4.6764 (PMC10807683; doi:10.1002/cam4.6764)
Supplement: Supplementary file 1 — Appendix S1. [file CAM4-13-e6764-s001.docx]

**Figure S1** Kaplan Meier curves for overall survival (A), progression-free survival (B) of patients receiving PD-1 inhibitors.


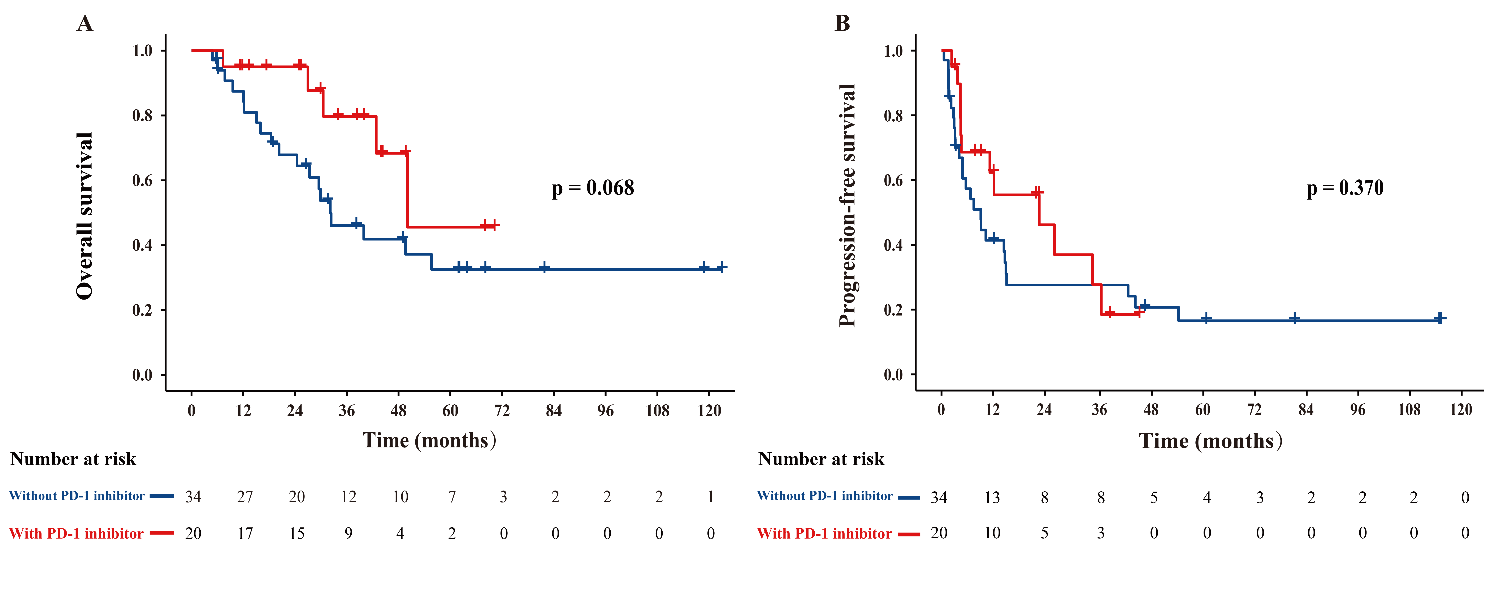


**Table S1** Systemic chemotherapy and PD-1 inhibitors

| **Treatment** | **No. of patients (%)** | **Dosing schedule per cycle** |
| --- | --- | --- |
| **Chemotherapy** |  |  |
| GP | 26 (48.1) | gemcitabine (1000 mg/m^2^, days 1, 8) plus cisplatin (80 mg/m^2^, day 1), every three weeks |
| TPF | 10 (18.5) | docetaxel (60-80 mg/m^2^, day 1) or paclitaxel (135-175 mg/m^2^ day 1), cisplatin (80 mg/m^2^, day 1) and 5-flurouracil (600-1000 mg/m^2^, days 1-5), every three weeks |
| TP | 2 (3.7) | docetaxel (60-80 mg/ m^2^, day 1) or paclitaxel (135-175 mg/ m^2^ day 1) and cisplatin (80 mg/ m^2^, day 1), every three weeks |
| PX | 9 (16.7) | cisplatin (80 mg/m^2^, day 1) plus capecitabine (1000 mg/m^2^, days 1-14), every three weeks |
| Others | 7 (13.0) | gemcitabine (1000 mg/m^2^, days 1, 8) plus vinorelbine (30 mg/m^2^, days 1, 8), every three weeks |
| **PD-1 inhibitors** |  |  |
| Camrelizumab | 15 (27.8) | 200 mg, day 1, every three weeks |
| Toripalimab | 1 (1.9) | 240 mg, day 1, every three weeks |
| Tirelizumab | 1 (1.9) | 200 mg, day 1, every three weeks |
| Sintilimab | 3 (5.6) | 200 mg, day 1, every three weeks |
| **Maintenance therapy** |  |  |
| Capecitabine | 12 (22.2) | 1000 mg/m^2^, twice daily on days 1-14, every 3 weeks |
| Camrelizumab | 8 (14.8) | 200 mg, day 1, every three weeks |
| Toripalimab | 3 (5.6) | 240 mg, day 1, every three weeks |
| Tirelizumab | 1 (1.9) | 200 mg, day 1, every three weeks |
| Sintilimab | 2 (3.7) | 200 mg, day 1, every three weeks |
| Anlotinib | 2 (3.7) | 12 mg, once daily on days 1-14, every 3 weeks |
| Apatinib | 1 (1.9) | 500 mg, once daily, every three weeks |

Abbreviations: GP, gemcitabine plus cisplatin; PD-1, programmed death-1; PX, cisplatin plus capecitabine; TP, docetaxel or paclitaxel; TPF, docetaxel or paclitaxel plus cisplatin and 5-flurouracil

**Table S2 Data on the timing of chemotherapy and SBRT.**

| Treatment Timing | Measurement | No. |
| --- | --- | --- |
| Sequencing | Concurrent Chemo-SBRT | 31 (57.4 %) |
|  | Upfront Chemo->RT | 20 (37.0%) |
|  | SBRT->Chemo | 2 (3.7%) |
|  | Missing | 1 (1.9%) |
| Diagnosis to the initiation of chemotherapy (months) | Median | 0.5 |
|  | Interquartile range | 0.2-1.1 |
|  | Range | 0-13.8 |
| Diagnosis to the initiation of SBRT (months) | Median | 6.1 |
|  | Interquartile range | 2.6-11.0 |
|  | Range | 0.6-24.9 |
| Initiation of chemotherapy to initiation of SBRT (months) | Median | 5.5 |
|  | Interquartile range | 1.8-9.7 |
|  | Range | -0.9-24.4 |

Abbreviations: SBRT, stereotactic body radiation therapy.

Table S3 Univariate and multivariate analysis of progression-free survival

| **Factor** | **Univariate analysis** | | |  | **Multivariate analysis** | | |
| --- | --- | --- | --- | --- | --- | --- | --- |
|  | **HR** | **95% CI** | **p value** |  | **HR** | **95% CI** | **p value** |
| Sex (male vs. female) | 2.61 | 1.01-6.75 | 0.047 |  | 4.71 | 1.58-14.02 | 0.005 |
| Age (≥ 46 vs. < 46 years) | 1.55 | 0.80-2.98 | 0.191 |  |  |  |  |
| ECOG (1 vs. 0) | 8.03 | 3.28-19.65 | < 0.001 |  | 3.10 | 1.14-8.45 | 0.027 |
| EBV-DNA (≤ 1000 vs. > 1000 copies/ml) | 0.47 | 0.24-0.91 | 0.026 |  | 0.39 | 0.19-0.79 | 0.009 |
| PD-1 inhibitors (yes vs. no) | 0.73 | 0.36-1.46 | 0.373 |  |  |  |  |
| Synchronous metastasis (yes vs. no) | 1.47 | 0.75-2.89 | 0.261 |  |  |  |  |
| Metastatic sites (single vs. multiple) | 0.44 | 0.22-0.86 | 0.017 |  |  |  |  |
| Lung metastasis (yes vs. no) | 1.10 | 0.56-2.19 | 0.779 |  |  |  |  |
| Liver metastasis (yes vs. no) | 1.20 | 0.63-2.29 | 0.575 |  |  |  |  |
| Bone metastasis (yes vs. no) | 2.74 | 1.31-5.75 | 0.008 |  |  |  |  |
| Oligometastases ^a^ (yes vs. no) | 0.19 | 0.08-0.42 | < 0.001 |  | 0.26 | 0.10-0.66 | 0.005 |
| Dose/fraction (≥ 8Gy vs. < 8 Gy) | 0.56 | 0.29-1.10 | 0.093 |  |  |  |  |
| Total dose (≥ 48 vs. < 48Gy) | 0.42 | 0.22-0.82 | 0.011 |  |  |  |  |
| BED (≥ 80 Gy vs. < 80Gy) | 0.37 | 0.19-0.72 | 0.003 |  |  |  |  |
| Time from metastasis diagnosis to SBRT (≤ 6 vs. > 6 months) | 0.33 | 0.16-0.65 | 0.001 |  | 0.28 | 0.13-0.61 | 0.001 |
| Cycles of systemic therapy (≤ 5 vs. > 5 cycles) | 0.72 | 0.38-1.38 | 0.324 |  |  |  |  |
| Maintenance therapy (yes vs. no) | 0.95 | 0.49-1.84 | 0.886 |  |  |  |  |

Abbreviations: BED, biological effective dose; EBV, Epstein-Barr Virus; ECOG, Eastern Cooperative Oncology Group; HR, Hazard Ratio; PD-1, programmed death-1; SBRT, stereotactic body radiation therapy.

a Oligometastases was defined as a limited metastatic spread (between 1 and 5 metastases) and low tumor burden.
